# Intravitreal Injections of Bevacizumab: The Impact of Needle Size in Intraocular Pressure and Pain

**DOI:** 10.5005/jp-journals-10028-1220

**Published:** 2017-08-05

**Authors:** Mónica Loureiro, Rita Matos, Paula Sepulveda, Dália Meira

**Affiliations:** 1 Resident, Department of Ophthalmology, Centro Hospitalar de Vila Nova de Gaia/Espinho, Porto, Portugal; 2 Resident, Department of Ophthalmology, Centro Hospitalar de Vila Nova de Gaia/Espinho, Porto, Portugal; 3 Consultant, Department of Ophthalmology, Centro Hospitalar de Vila Nova de Gaia/Espinho, Porto, Portugal; 4 Consultant, Department of Ophthalmology, Centro Hospitalar de Vila Nova de Gaia/Espinho, Porto, Portugal

**Keywords:** Bevacizumab, Intravitreal injections, Intraocular pressure, Pain, Visual analog scale.

## Abstract

**Aim:**

To compare the effect of 30-gauge *vs* 27-gauge needle size on intraocular pressure (IOP) rise and patients’ pain experience after intravitreal injection (IVI) of bevacizumab.

**Materials and methods:**

Cross-sectional, randomized, double-armed study. Patients were randomized to IVI with 30-gauge or 27-gauge needle. The IOP was measured pre and post IVI. Patients’ pain was graded using the visual analog scale (VAS).

**Results:**

A total of 54 eyes were included. The IVI caused a significant IOP rise in both groups (p < 0.001). In the 30-gauge group, the mean pre- and postinjection IOP was 16.3 ± 3.6 mm Hg and 24.1 ± 9.0 mm Hg. The corresponding figures in the 27-gauge group were 18.0 ± 2.54 (p = 0.26) and 23.1 ± 7.5 mm Hg (p = 0.66). In the 30-gauge group, the mean VAS pain score was 3.2 ± 2.6 compared to 3.0 ± 2.5 in the 27-gauge group (p = 0.78).

**Conclusion:**

The IVI caused a significant rise in IOP after the injection, independently of the needle size used. The 27-gauge needle coursed with lower postinjection IOP without prejudice of the patient comfort.

**Clinical significance:**

The IVI with 27-gauge may be considered for glaucomatous eyes (higher risk eyes), for which IOP spikes are not recommended.

**How to cite this article:**

Loureiro M, Matos R, Sepulveda P, Meira D. Intravitreal Injections of Bevacizumab: The Impact of Needle Size in Intraocular Pressure and Pain. J Curr Glaucoma Pract 2017;11(2):38-41.

## INTRODUCTION

The intravitreal injection (IVI) of anti-vascular endothelial growth factor (anti-VEGF) has become one of the most common intraocular procedures over the past decade.^1^ The efficacy and safety profile of anti-VEGF therapy have revolutionized the treatment of several retinal diseases, such as neovascular age-related macular degeneration, retinal vein occlusion, and diabetic macular edema.^2-4^ Bevacizumab, an off label anti-VEGF drug used worldwide in the clinical practice for the last decade, is an humanized monoclonal antibody that binds all VEGF isoforms and interferes with receptor binding to inhibit its signal.^3^ Its efficacy and safety profile are similar to ranibizumab, but much less expensive, and for this reason is usually the first treatment choice.^5^

The majority of patients, over the course of their diseases, require multiple anti-VEGF IVI. The intraocular pressure (IOP) rise occurring after this procedure revealed to be transient and usually normalizes spontaneously within 30 minutes;^6-8^ however, repeated IOP spikes may be a contributing factor for sustained IOP elevation in susceptible eyes, related to the trabecular meshwork damage.^8,9^ The presence of vitreous reflux has been associated with lower postinjection IOP^10^ and Pang et al^11^ reported that the incidence of vitreous reflux was higher with larger bore size needles. Hence, we hypothesize that larger 27-gauge needles are less associated with IOP spikes, as they may cause higher vitreous reflux.

Despite this, there is a tendency to favor smaller bore size needles owing to the requirement for less force to penetrate the sclera and the belief that it induces less pain.^12^ The patient’s pain experience during the injection may hinder his collaboration with sudden movements of the eye that can potentially cause complications and may also affect the decision to keep the treatments. Therefore, patients’ comfort has to be taken into account, as IVI treatments are rising exponentially. The visual analog scale (VAS) is a common and easy tool for assessing pain and it has been shown to be a valid and reliable research method in previous ophthalmology studies.^13-15^

The main purpose of the study was to compare the effect of size on immediate postinjection IOP and on patients’ pain experienced after the IVI of bevacizumab.

## MATERIALS AND METHODS

The study was approved by the local ethics committee and informed consent (according to the tenets of the Declaration of Helsinki) was obtained from participating patients. All patients, referred for IVI of bevacizumab by an ophthalmologist from our institution, older than 18 years of age and mentally able to score the pain, were selected to participate in the study. The exclusion criteria were:

- Previous ocular surgery other than cataract extraction with posterior chamber intraocular lens
- Use of lowering IOP drugs
- Corneal diseases that might interfere with the tonometry and
- Ocular pain prior to the procedure.

The patients were randomly selected to be injected with a 30-gauge (0.40 × 12 mm, Braun®) or a 27-gauge (0.30 × 12 mm, Braun®) needle. The periocular skin, eyelid margins, and eye lashes were cleaned with 10% povidone iodine before the IVI. After topical anesthetic oxibu-procaine 0.4% and topical 5% povidona-iodine solution application to the conjunctiva, an eyelid speculum was positioned to stabilize the eyelids. No sedation was administered to any patient. Bevacizumab (1.25 mg/0.05 mL) was dispensed into single-use 27-gauge and 30-gauge needle syringes using an aseptic technique. The anti-VEGF was injected into the vitreous cavity using a straight scleral technique through the pars plana, 3.5 to 4.0 mm posterior to the limbus at the superotemporal quadrant, with the patient in supine position and under sterile conditions.

All patients had immediate pre- and postinjection IOP measurements, using Goldmann applanation tonometry, by the same ophthalmologist who was masked to the needle caliber. The measurements were performed three times and the mean value was used for the analysis. The postinjection IOP was consistently measured within the first 5 minutes after the IVI. After the injection, patients were also asked to rate their pain experience during the procedure on a VAS, which is a psychometric response scale for subjective characteristics that cannot be directly measured, indicating a position along a continuous line between two end points 0 and 10. All VAS measurements were collected by a single ophthalmologist after explaining this method to the patients.

Demographic and patients’ characteristics were compared using descriptive statistics and univariate analysis as appropriate. Immediate postinjectionIOP and acute IOP variation between groups were compared by a 2-tailed t test. Considering the VAS scores, categorical parameters were analyzed using Mann-Whitney test and continuous parameters were analyzed by Spearman’s rank correlations. A value of P < 0.05 was considered to indicate statistical significance.

**Graph 1:**
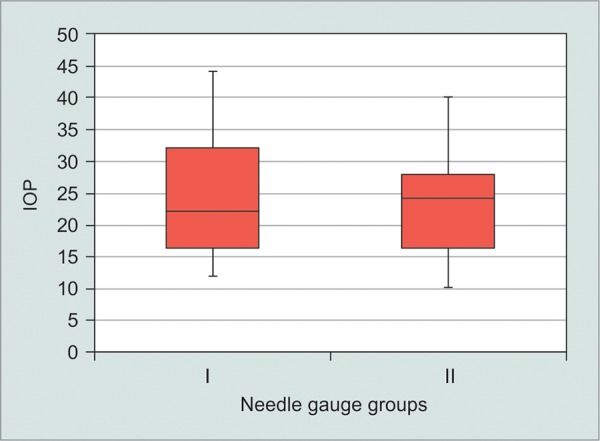
The postinjection IOP (within the 1st minutes) using 30-gauge needle (group I) and 27-gauge needle (group II)

**Graph 2:**
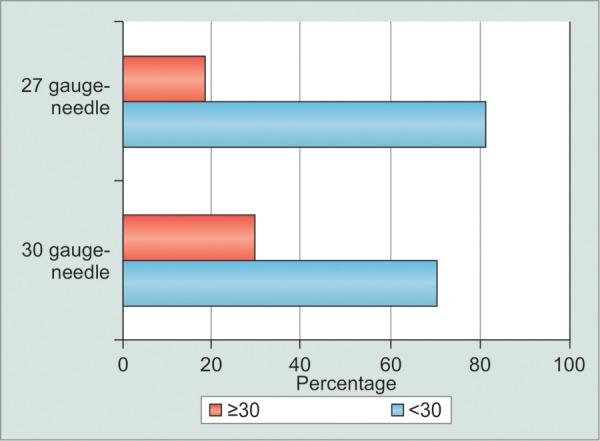
The percentage of eyes with postinjection IOP of 30 mm Hg or higher after injection using 30-gauge and 27-gauge needles

## RESULTS

Fifty-four eyes of 48 patients received the bevacizumab IVI for the treatment of diabetic macular edema (63%), neovascular age-related macular degeneration (26%), and retinal vein occlusion (11%). Half of them were injected with 27-gauge needles whereas the other half with 30-gauge needles.

In the 30-gauge group, the mean age was 68.7 ± 9.8 years (54-87) and the mean pre-IVI IOP was 16.3 ± 3.6 mm Hg. In the 27-gauge group, the mean age was 70.1 ± 10.4 years (47-86) and the mean pre-IVI IOP was 18.0 ± 2.5 mm Hg. The groups were compared using independent sample t-test and were matched for age (p = 0.44) and pre-IVI IOP (p = 0.26). The mean number of IVI previously performed in each patient was 5.3 ± 2.9 in the 30-gauge group compared to 4.3 ± 3.6 in the 27-gauge group (p = 0.32).

After IVI, a significant rise of the IOP occurred within the first 5 minutes in both groups (p < 0.001) and the mean increase was of 6.9 ± 8.2 mm Hg. Eyes injected with 30-gauge needles presented a mean post-IVI IOP of 24.1 ± 9.0 mm Hg, whereas the corresponding figure in the 27-gauge group was lower: 23.1 ± 7.5 mm Hg (p = 0.66) (Graph 1). The incidence of a post-IVI IOP of 30 mm Hg or higher was 29.6% (n = 8) using 30-gauge needles compared to 18.5% (n = 5) using 27-gauge needles (p = 0.34) (Graph 2). The acute IOP variation, representing the difference between the pre- and postinjection IOP, was 7.9 ± 9.1 mm Hg in eyes injected with 30-gauge needles, compared to 5.9 ± 7.2 mm Hg in the 27-gauge needles group (p = 0.38, 2-tailed t-test).

**Graph 3:**
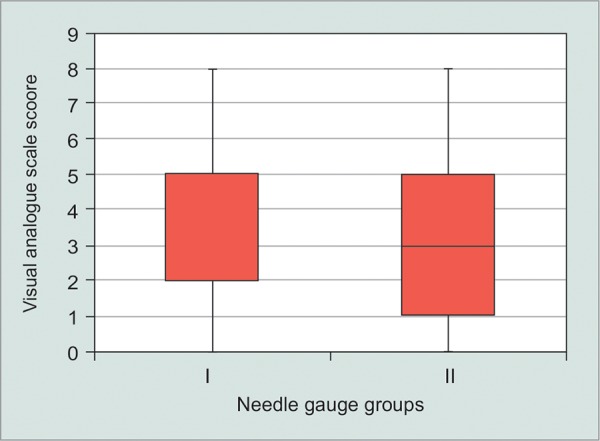
The distribution of VAS scores using 30-gauge needle (group I) and 27-gauge needle (group II)

The mean VAS pain score in the 30 gauge needle and 27 gauge needle groups was 3.2 ± 2.6 (median 2, mode 2) and 3.0 ± 2.2 (median 3, mode 1) respectively. No significant difference was demonstrated between the two groups regarding pain during the injection (p = 0.78) (Graph 3). No significant correlation was found between pain scores and patient’s age (R = 0.31) or previous IVI number (R = -0.12).

## DISCUSSION

The IVI of anti-VEGF is a widespread accepted procedure; however, there is no clear consensus in terms of needle gauge used.^9,16^ The arguments surrounding the appropriate needle size choice have been related to the patients’ pain experience during the injection and the immediate post-IVI IOP. Therefore, this study might contribute to clarify the significance of needle choice, taking into account these two parameters, and thus helping to establish a standardized protocol.

Our study evidenced a significant IOP rise within the first minutes after the IVI, independently of the needle bore size. Despite the fact that the 27-gauge group presented a lower acute IOP variation, there was not a significant difference compared to the 30-gauge group. Previously, Kim et al^17^ showed greater IOP with 30- and 32-gauge needles in comparison to 27-gauge needles; however, the injections done with 30- and 32-gauge needles consisted of bevacizumab or ranibizumab, while injections done with 27-gauge needles were of pegaptanib or triamcinolone. Differently, in our study, we used the same drug and volume in both groups.

Pang et al^11^ investigated the effect of needle size on post-IVI IOP and reflux and found that eyes injected with 32-gauge needles had higher IOP and lower incidence of vitreous reflux compared to the eyes injected with larger 30-gauge needles. Therefore, these findings suggest that a larger bore size needle facilitate the vitreous reflux and thus lower IOP spikes after IVI are expected. In fact, in our study, the 27-gauge group had lower IOP after the injection and, furthermore, the IOP variation was lower than with 30-gauge needles, however, not significantly.

Attending to the technique, the tunneled scleral and the straight scleral IVI were previously compared by Knecht et al^18^, in terms of IOP increase after the injection and patient discomfort, without differences between the groups. Therefore, we used a straight scleral IVI technique in all patients and only studied the needle size impact.

With the increasing occurrence of patients receiving injections, often repeated for several times, evaluating the pain associated with this procedure is important to optimize patient’s comfort and compliance. The VAS is a convenient tool, easily performed by anyone cognitively capable of understanding the parameters and responding to clinician instruction. After a brief explanation, no patients had difficulty in classifying the degree of their pain. The VAS is frequently used in the Portuguese population to assess the analgesic effect of a therapy and has been widely used in ophthalmic research.^13-15^ In our study, the IVI proved to cause only a mild pain, being well tolerated by the patients, independent of the needle bore size used.

Regarding the caliber of the needle and the pain during IVI, Guler et al^19^ and Rodrigues et al^20^ reported that patients injected with 30-gauge needle experienced less pain compared to those injected with 27-gauge needle. In fact, Pulido et al^12^ reported that 27-gauge needles require almost twice the force to penetrate the sclera, which theoretically may have implications for patients’ comfort. However, both Rifkin and Schaal^21^ and Haas et al^22^ found that the 27- and 30-gauge needles did not influence significantly the pain. In a similar way, in our study, there was no difference between the 27- and 30-gauge needles.

A limitation of our study was the sample size which did not allow to analyze the effect of demographic factors on the pain score and postinjection IOP. Although the evaluation of the vitreous reflux could be of interest, the assessment of its absence (no detectable reflux) or presence (any visible reflux) is subjective. Therefore, quantitative studies regarding vitreous reflux and post-IVI IOP are needed. The axial length was not taken into account; however, it was not a predictor of subsequent IOP elevation in a previous study.^23^

## CONCLUSION

The IVI caused a significant rise in IOP after the injection, independently of the needle size used. Although not significantly, the postinjection IOP and IOP variation were lower in the 27-gauge group. The 27-gauge might be selected in cases requiring less IOP variation, such as glaucomatous eyes in which IOP spikes can cause visual field deterioration. The use of 27-gauge needles showed no significant effect in the patients’ pain level.
